# Review of evolving etiologies, implications and treatment strategies for the superior vena cava syndrome

**DOI:** 10.1186/s40064-016-1900-7

**Published:** 2016-02-29

**Authors:** Christopher Straka, James Ying, Feng-Ming Kong, Christopher D. Willey, Joseph Kaminski, D. W. Nathan Kim

**Affiliations:** Department of Radiation Oncology, University of Texas Southwestern Medical Center, 5801 Forest Park Rd, Dallas, TX 75390 USA; Department of Radiation Oncology, GRU Cancer Center and Medical College of Georgia, Augusta, GA USA; Department of Radiation Oncology, Texas Oncology, 1700 W. Highway 6, Waco, TX 76712 USA; Department of Radiation Oncology, The University of Alabama Birmingham, Birmingham, AL USA; Dattoli Cancer Center, 2803 Fruitville Rd, Sarasota, FL 34237 USA

**Keywords:** Superior vena cava syndrome (SVC syndrome, SVCS), Stenting, Thoracic malignancies, Hypo-fractionation, Multi-modality therapy

## Abstract

Superior vena cava syndrome (SVCS) is a relatively common sequela of mediastinal malignancies and may cause significant patient distress. SVCS is a medical emergency if associated with laryngeal or cerebral edema. The etiologies and management of SVCS have evolved over time. Non-malignant SVCS is typically caused by infectious etiologies or by thrombus in the superior vena cava and can be managed with antibiotics or anti-coagulation therapy, respectively. Radiation therapy (RT) has long been a mainstay of treatment of malignant SVCS. Chemotherapy has also been used to manage SVCS. In the past 20 years, percutaneous stenting of the superior vena cava has emerged as a viable option for SVCS symptom palliation. RT and chemotherapy are still the only modalities that can provide curative treatment for underlying malignant etiologies of SVCS. The first experiences with treating SVCS with RT were reported in the 1970’s, and several advances in RT delivery have subsequently occurred. Hypo-fractionated RT has the potential to be a more convenient therapy for patients and may provide equal or superior control of underlying malignancies. RT may be combined with stenting and/or chemotherapy to provide both immediate symptom palliation and long-term disease control. Clinicians should tailor therapy on a case-by-case basis. Multi-disciplinary care will maximize treatment expediency and efficacy.

## Background

Superior vena cava syndrome (SVCS), a clinical manifestation arising from compression of the thin-walled superior vena cava (SVC), was first described by William Hunter in 1757 and can be caused by a variety malignancies (Hunter and Johnston 1757). SVCS is a significant disorder affecting up to 10 % of small cell lung cancer (SCLC) patients and 2–4 % of all lung cancer patients (Wan and Bezjak 2010). The prognosis of SVCS caused by malignancies is primarily determined by the tumor type (Yu et al. 2008). Traditionally, malignant SVCS has been considered to be an indication for emergent intervention, typically with radiation therapy (RT). However, accumulating evidence has suggested that accurate diagnosis and biopsy should precede emergent therapeutic intervention in most cases (Yu et al. 2008). Timely and effective intervention aimed at treating the malignant cause of this syndrome can relieve significant suffering and improve SVCS patients’ quality of life.

## Anatomy and physiology

The SVC carries approximately one-third of the venous return to the heart. Situated slightly to the right of midline and coursing through the superior and middle mediastinum, the SVC is the major drainage outlet for venous return from the head, arms, and upper torso. Despite its high flow volume, the SVC is quite distensible and can be compressed by an adjacent mass originating in the middle or anterior mediastinum, the right paratracheal or precarinal lymph node stations, or the right lobe bronchus (Wan and Bezjak 2010; Wilson et al. 2007). Right-sided masses are more likely to cause SVCS, presumably due to the anatomic location of the SVC (Sculier et al. 1986). Rarely, a thrombus can occlude the SVC even in the absence of an external mass.

The superior and middle mediastinum is an anatomically confined space populated by a large number of lymph nodes. The SVC is thin walled and is opened by relatively low venous pressure, making it particularly susceptible to compression by adjacent masses (Koetters 2012). In the event of SVC obstruction, venous pressure in collateral vessels increases and, over time, a collateral blood-flow network develops (Lacout et al. 2012). Many different vessels may enlarge in response to the increased blood pressure, including the azygos, the hemiazygos, intercostal, mediastinal, paravertebral, thoracoepigastric, internal mammary, thoracoacromioclavicular, and anterior chest wall veins (Eren et al. 2006). The azygos vein may connect the SVC and the inferior vena cava (IVC) directly, so SVC obstructions below the insertion of the azygos vein typically result in more severe symptoms of SVCS (Stanford et al. 1987). Typically, multiple weeks are required for development of sufficiently large collateral network to accommodate the blood flow that normally passes through the SVC (Kim et al. 1993; Trigaux and Van Beers 1990). If SVC occlusion occurs relatively slowly, then patients may remain asymptomatic as the collateral network is able to grow apace with the increasing SVC obstruction. In contrast, rapidly developing, complete or near-complete SVC obstructions can cause a variety of sequelae, including dyspnea; swelling of the neck, trunk, upper extremities and face; chest pain; collateral venous distension; plethora; dysphagia; and hoarseness (Armstrong et al. 1987; Rice et al. 2006; Chen et al. 1990; Mineo et al. 1999).

Increased venous pressure can lead to marked edema of the upper body including the face, neck, upper extremities. This can cause significant patient discomfort and, in severe cases, narrowing of the upper respiratory tract secondary to nasal and laryngeal edema (Baker and Barnes 1992). Rarely, cerebral edema may develop and necessitate emergent treatment (Baker and Barnes 1992; Sofue et al. 2013; Taguchi et al. 2011). Death is very rarely caused by SVCS. In one series of 1986 patients with SVCS, only 1 death was reported (Ahmann 1984).

## Etiologies

Common causes for masses that can impinge on the SVC include enlarged paratracheal lymph nodes of malignancies, lymphoma, thymoma, inflammatory processes, and aortic aneurysms. Before antibiotics came into widespread use, infectious etiologies were a common cause of SVCS. Specifically, aortic aneurysms due to tertiary syphilis were frequent (Schechter 1954). More recently, however, tertiary syphilis has become rare, while the overall incidence of malignancies has risen due to the increased average lifespan of our population. In the 1970s and 1980s, malignancies caused 78–93 % of SVCS (Chen et al. 1990; Parish et al. 1981). In the past 20 years, intravascular devices such as catheters and pacemakers have become much more common (Cheng 2009). Thrombi associated with these devices have emerged as a significant cause of SVCS, accounting for up to 28 % of cases of SVCS in some reports (Rice et al. 2006a; b). Despite the recent rise in thrombus-driven SVCS, malignancies remain the most common cause of SVCS. Furthermore, we may presume that, as surgical experience and expertise with implantable devices matures, the majority of SVCS will continue to be malignancy-derived and the percentage of SVCS due to malignancy may actually rise in subsequent decades. As seen in Table 1, among the malignant causes of SVCS, non-small cell lung cancer (NSCLC) (22–57 %), small-cell lung cancer (SCLC) (10–39 %), and lymphoma (1–27 %) are by far the most common causes (Armstrong et al. 1987, 2006; Chen et al. 1990; Yellin et al. 1990; Schraufnagel et al. 1981; Nicholson et al. 1997; Hohloch et al. 2014).

**Table 1:** Etiologies of superior vena cava syndrome

Etiology (Rice et al. 2006a; Chen et al. 1990; Mineo et al. 1999; Schraufnagel et al. 1981; Nicholson et al. 1997; Hohloch et al. 2014; Lonardi et al. 2002)	Prevalence (%) (Rice et al. 2006a; Chen et al. 1990; Mineo et al. 1999; Schraufnagel et al. 1981; Nicholson et al. 1997; Hohloch et al. 2014; Lonardi et al. 2002)
*Malignant*	
Non-small cell lung cancer	22–57
Small cell lung cancer	10–39
Lymphoma	1–27
“Other metastasis”	19
Other adenocarcinoma	3–15
Germ cell tumors	2–6
Thymoma	1–3
Sarcomas	2
Esophageal carcinoma	2
AML	1
Tuberculosis lymphangitis	2
*Benign*	
Port-a-cath	16
Dialysis catheter	5
Fibrosing mediastinitis	2–9
Mesothelioma	1–7
Mustard operation	5
Primary SVC thrombosis	1–5
Retrosternal goiter	3
Tuberculosis lymphangitis	2
Behcet’s syndrome	2
Pacemaker wire	1–2
Hematoma after aortic dissection repair	1
Pseudotumor	1
Hickman catheter	1
Aneurysm	1
Radiation fibrosis	1

Malignant and non-malignant causes of superior vena cava syndrome

## Clinical evaluation

SVCS presents with signs and symptoms that may be readily identified on clinical evaluation (Cheng 2009). The most common symptoms of SVCS include neck swelling (100 %), dyspnea (54–83 %), swelling of the trunk and/or upper extremities (38–75 %), facial swelling (48–82 %), chest pain (15 %), cough (22–58 %), dilated chest vein collaterals (38 %), weight loss (10–31 %), jugular venous distension (27 %), phrenic nerve paresis (16.2 %), plethora (13 %) and dysphagia (10–13 %) (Armstrong et al. 1987; Rice et al. 2006a; Chen et al. 1990; Mineo et al. 1999). More rarely reported symptoms include hoarseness, headache, confusion, dizziness, night sweats, hypoxia, hyponatremia, and syncope (Wan and Bezjak 2010; Rice et al. 2006a; Mineo et al. 1999). Symptoms typically have a gradual onset. In general, the faster the onset of symptoms the more severe the symptoms, as slowly advancing obstruction of the SVC allows time for collateral circulation development.

The most common findings on physical exam include edema of the face, neck, trunk, and upper extremities as well as collateral venous distension of the neck and the anterior chest wall. More rarely, plethora or cyanosis can be observed. Symptoms may include dyspnea, dysphagia, hoarseness, stridor, cough, and chest pain. Symptoms associated with cerebral edema can include neurologic symptoms such as headaches, confusion, dizziness, obtundation, and mental status changes (Yu et al. 2008).

To assist in determining the urgency of intervention and tracking progression of symptoms, a classification scheme has been proposed for grading SVCS according to symptom severity (Lonardi et al. 2002). This classification schema includes parameters such as degree of cerebral edema, laryngeal edema, and hemodynamic compromise to differentiate between life-threatening (grade 4), severe (grade 3), and non-life threatening (grade 0–2) (Yu et al. 2008). Another scaled scoring system, the Kishi score, has been developed to assist in making the decision to initiate stent therapy. The Kishi score system incorporates neurological, laryngeal, facial, and cardiovascular signs and symptoms (Kishi et al. 1993). As seen in Table 2, a score of 4 or higher using the Kishi scoring system indicates a need for percutaneous stenting.

**Table 2:** Kishi Scoring system

Clinical signs (Lacout et al. 2012; Kishi et al. 1993)	Weighting
*Neurological signs*	
Awareness disorders or coma	4
Visual disorders, headache, vertigo or memory disorders	3
Mental disorders	2
Malaise	1
*Thoracic or pharyngeal*-*laryngeal signs*	
Orthopnea or laryngeal edema	3
Stridor, dysphagia or dyspnea	2
Coughing or pleurisy	1
*Facial signs*	
Lip edema, nasal obstruction or epistaxis	2
Facial edema	1
*Vessel dilation*	
Neck, face or arms	1

Presence of any of the symptoms in the left column give the points indicated in the right column. The total points are added to together. A score of 4 or higher indicates a need for percutaneous stent placement

## Imaging

Chest imaging is an important diagnostic tool that can frequently be used to find the abnormality underlying SVCS (Lacout et al. 2012). 84 % of SVCS patients have abnormal chest X-rays, with 64 % demonstrating widening of the superior mediastinum and 26 % demonstrating pleural effusion (Parish et al. 1981). Ultrasound can be used to rule out venous thrombus, but, due to the interposition of overlying ribs between the probe and the mediastinum, ultrasound cannot be used to directly image the SVC. However, Doppler ultrasound can reveal flow reversal in the internal thoracic vein, which is indicative of SVC obstruction (Spiro et al. 1983). Similarly, Doppler ultrasound can show a resolution of blockage and return to normal flow after successful treatment (Lacout et al. 2012). Computed tomography (CT) scans and magnetic resonance imaging (MRI) studies are commonly used in initial evaluation of SVCS and can reveal SVC blockage prior to symptom development (Uberoi 2006).

Contrast-enhanced spiral or multi-slice CT imaging may be used to elucidate the cause as well as the extent of venous obstruction. These imaging modalities may reveal distended collateral vasculature, which strongly suggest SVCS with sensitivity of 96 % and specificity of 92 % (Kim et al. 1993). Contrast-enhanced CT imaging can also be used to elucidate physical characteristics of the obstruction in preparation for stent insertion (Spiro et al. 1983). A classification schema of SVC stenosis based on CT findings has been proposed, and it may be useful for determining prognosis and utility of stent insertion (Lacout et al. 2012). If higher resolution imaging is needed, multi-detector CT can be used to provide 3-dimensional anatomic detail of collateral vasculature (Eren et al. 2006). Lastly, for patients who cannot tolerate the long duration of MRI, CT venography may be considered. Complications of venography are rare and mild, including bleeding from the site easily controlled by a few minutes of direct pressure (14 %) (Rice et al. 2006a) and transient respiratory distress (0.5 %) (Ahmann 1984).

Contrast-enhanced magnetic resonance (MR) venography is a highly sensitive and accurate diagnostic modality, shown to be up to 100 % sensitive and specific for diagnosing central venous abnormalities (Davenport et al. 1976; Rachapalli and Boucher 2014). Unfortunately, it is expensive and time-consuming, and dyspneic SVCS patients may have difficulty remaining supine for the entire imaging process. For patients allergic to contrast dye or with difficult venous access, standard MRI is often sufficient (Cheng 2009).

Superior vena cavography is generally carried out before stenting, and is considered the gold standard for detecting thrombotic obstruction in the SVC and demonstrating the extent of thrombus formation (Uberoi 2006). It has been shown to be better than conventional CT for visualizing opacified collateral vessels, extension of thrombi into peripheral vessel, and the degree of obstruction (Cheng 2009). It is less useful for revealing causes of obstruction other than thrombus.

## Histology

Imaging may distinguish between masses and thrombi as causes of SVCS, but, in the case of an obstructing mass where suspicion for malignancy is high, biopsy and histological studies are required to determine the type of malignancy. One study revealed that up to 59 % of SVCS patients present without a previous cancer diagnosis (Schraufnagel et al. 1981). Minimally invasive techniques such as sputum cytology, pleural fluid cytology, or superficial lymph node biopsy can be used to diagnose up to two-thirds of malignancies (Schraufnagel et al. 1981). To diagnose the remaining one-third, invasive procedures such as bronchoscopy, mediastinoscopy, mediastinotomy, thoracotomy, and thoracoscopy may be required to classify primary tumor type. Bone marrow biopsies may be able to provide a diagnosis as well as determine the stage of malignant lymphoma, and, less commonly used in current medical practice, small cell lung cancer. Most studies report low rates of complication and high diagnostic value in mediastinal procedures (Rice et al. 2006a; Mineo et al. 1999). Major bleeding and respiratory distress rarely occurred after thoracotomy, mediastinoscopy, or bronchoscopy (Ahmann 1984).

## Management

**Fig. 1:**
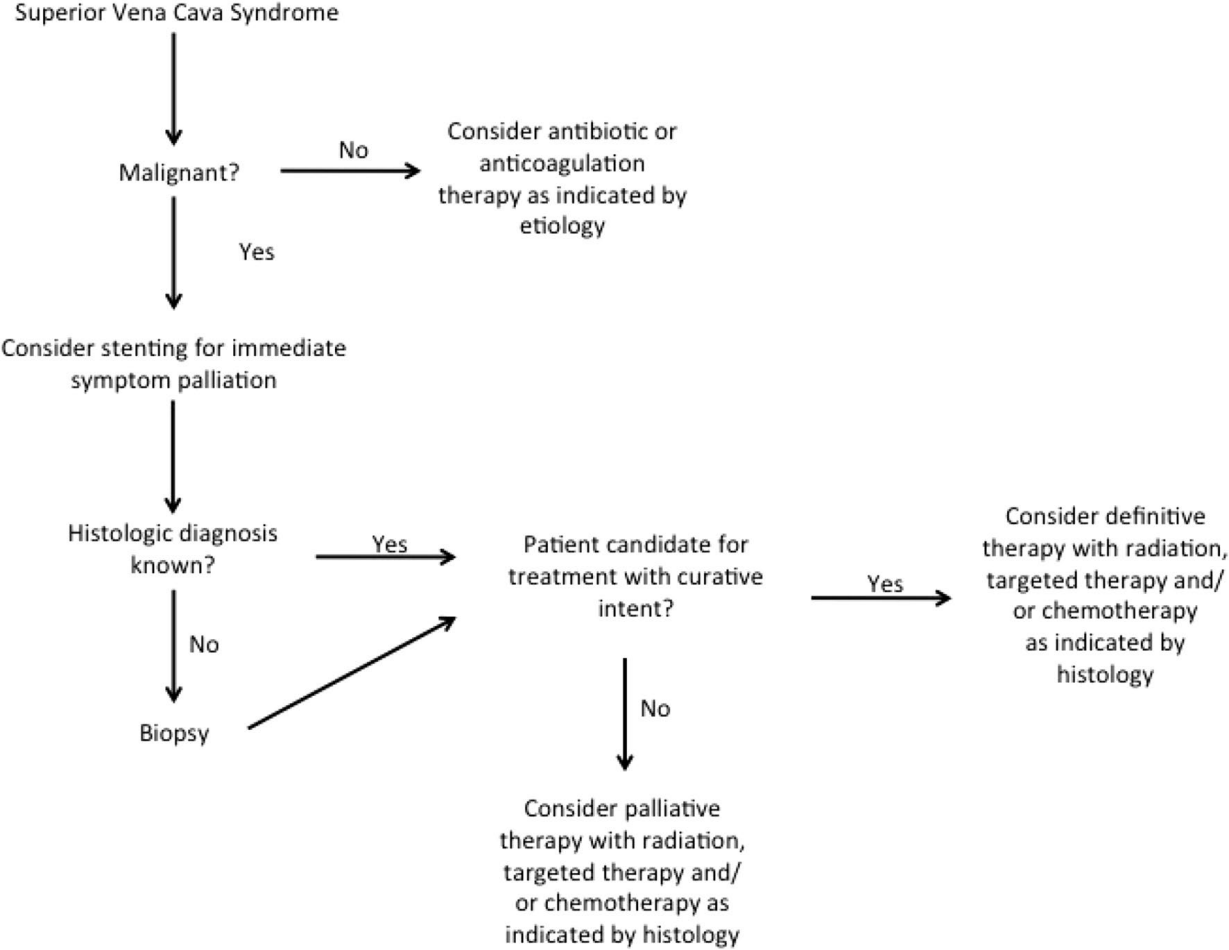
Management algorithm for SVCS. A broad overview that may be used to guide clinical decision making

Figure 1 provides a management algorithm that may be used by practitioners to guide clinical decision making.

### Emergent palliation

In select cases, SVCS may be life threatening and requires emergent treatment. If laryngeal edema causing laryngeal constriction or cerebral edema is present, these medical emergencies require prompt management and rapid treatment of the underlying cause of SVCS may be indicated (Baker and Barnes 1992; Sofue et al. 2013; Taguchi et al. 2011). These clinical SVCS sequelae may cause long-term morbidity or mortality if left untreated, and empiric treatment with radiation, stenting, and/or chemotherapy may be indicated even before biopsy results become available. Similarly, if clinical and radiographic evidence reveals a rapidly growing malignancy with a high likelihood of invading other critical thoracic structures, then prompt treatment to retard cancer growth is indicated (Devita Jr 1997). To minimize respiratory and cardiac complications in patients who present with tracheal obstruction or pericardial effusion, stenting and immediate RT may be recommended before attempting invasive procedures (Sculier et al. 1986).

As mentioned above, multiple scoring systems have been developed to assist practitioners in classifying the urgency of SVCS cases. In Yu et al.’s classification scheme, grade 4 SVCS indicates life-threatening disease due to one or more of the following: “significant cerebral edema (confusion, obtundation) or significant laryngeal edema (stridor) or significant hemodynamic compromise (syncope without precipitating factors, hypotension, renal insufficiency).” Although only 5 % of SVCS patients present with grade 4 disease, any of the aforementioned complications would be an indication for emergent venogram, stent placement, and thrombolytic therapy if indicated (Yu et al. 2008). Of note, immediate RT is not indicated as first line treatment in emergent cases of SVCS if stenting is feasible, as stenting has been shown to provide faster symptom resolution (Nicholson et al. 1997).

### Urgent palliation

Previously, all case of SVCS were considered medical emergencies. More recently, numerous reviews have shown no association between symptom duration and long-term treatment outcomes for most cases of SVCS when using chemotherapy, radiation, or stenting (Schraufnagel et al. 1981; Mose et al. 2006; Khan et al. 2014; Gauden 1993; Ampil et al. 2012). Furthermore, obtaining an accurate histologic diagnosis prior to starting RT allows for optimum treatment of the causative malignancy (Ampil et al. 2012; Kvale et al. 2007).

For SVCS caused by an infectious etiology, antibiotics should be the first line of treatment. In cases of thrombus-driven SVCS, anticoagulation therapy is first-line treatment and may effective in up to 88 % of patients if anticoagulation is initiated within 5 days of thrombus development (de Jager et al. 2013; Gray et al. 1991). Some experts recommend continuing anti-coagulation therapy indefinitely after an incident of SVC occlusion by a thrombus (Cheng 2009).

Although they have no effect on the underlying etiology of SVCS, several maneuvers are available for symptom palliation in significantly distressed patients. The underlying goal of these maneuvers is to reduce hydrostatic pressure in the upper half of the body. To that end, the patient should assume a seated position or at least elevate his head (Cheng 2009). Additionally, fluid restriction, diuretics and/or supplemental oxygen may be considered (Wan and Bezjak 2010; Wilson et al. 2007).

Radiation with or without concomitant steroids or chemotherapy was long considered to be the quickest and most efficacious option for long-term symptom relief. SVCS symptom improvement may begin as early as 3–9 days after starting RT, although response times of more than 30 days have been reported (Armstrong et al. 1987; Yellin et al. 1990; Schraufnagel et al. 1981; Nicholson et al. 1997; Mose et al. 2006; Ostler et al. 1997; Davenport et al. 1978). In the past decade, advances in interventional radiology have brought percutaneous stenting to the forefront of SVCS care. Placement of a percutaneous stent can give symptom relief starting as soon as 0–72 h after the procedure (Watkinson et al. 2008; Hennequin et al. 1995; Rosch et al. 1992). As detailed in Table 3, RT, chemotherapy, and stenting all offer unique advantages and disadvantages for SVCS patients, and practitioners should choose the most appropriate treatment modality based on the individual case presentation.

**Table 3:** Comparison of treatment modalities

	Time to symptom relief	% Chance of partial symptom relief	Can be combined with other therapies?	Treatment-associated mortality
Radiation Therapy	3–30 days (Armstrong et al. 1987; Mose et al. 2006; Ostler et al. 1997; Davenport et al. 1978; Rodrigues et al. 1993)	56–96 (Armstrong et al. 1987; Rodrigues et al. 1993)	Yes	Low
Chemotherapy	1–2 weeks (Rowell and Gleeson 2001)	59–77 (Rowell and Gleeson 2001)	Yes	Low
Stent placement	0–72 h (Hennequin et al. 1995; Rosch et al. 1992)	80–95 % (Uberoi 2006)	Yes	3–4 % (Uberoi 2006)

Properties of various treatment modalities used in superior vena cava syndrome

## Radiotherapy

### Palliative radiotherapy

RT has long been considered a mainstay of SVCS treatment (Wan and Bezjak 2010; Wilson et al. 2007; Ostler et al. 1997; Egelmeers et al. 1996). Although stent placement has recently emerged as a viable palliative option in many case of SVCS, RT remains an important part of treatment in most cases of SVCS and may be used as sole treatment in select cases.

RT typically does not achieve complete relief of SVC obstruction. In one review, post-RT venography showed a normal SVC flow in only 11.1 % of patients, and post-mortem analysis demonstrated completely or partially patent SVCs in only 24.2 % of patients. Despite this, the clinical response rate in this cohort was roughly 50–70 % (Ahmann 1984). Decreased tumor bulk after RT probably results in an increased capacity for collateral circulation, and may account for the discrepancy between symptom improvement and SVC re-patency after RT (Wilson et al. 2007).

The first experiences with RT for SVCS were published in the 1970s. Relatively high daily doses of 400 centiGray (cGy) to the mediastinum were delivered for the first 3 days followed by 150 cGy fractions to a total of 3000–5000 cGy (Davenport et al. 1976). Patients reported a subjective 77 and 91 % response rate at 3–4 and 7 days after treatment, and an objective response rate of 66 and 89 % was observed 3–4 and 7 days after treatment (Davenport et al. 1978). Given the high response rate and the relatively rapid time to symptom palliation, RT became the treatment of choice for patients with SVCS.

A retrospective study of 125 SVCS patients treated with RT from 1965 to 1984 found that 83 % of patients receiving high initial dose RT responded to treatment while 78 % of patients receiving conventional initial dose therapy responded to treatment. 70 % of patients treated with high initial dose RT (defined in this study as 300–400 cGy for 3 fractions at the beginning of therapy) showed a response within 2 weeks while only 56 % of patients receiving conventional fractionation RT up front experienced a response in that timeframe, but the difference was not statistically significant. Lastly, a dose-dependent response to RT was observed. Only 50 % of patients receiving less than 20 Gy responded to therapy, while 87 % of patients receiving greater than 20 Gray (Gy) responded to treatment. Dysphagia, the most common side effect, was experienced by 26 % of patients. Only 13 % of patients experienced recurrent SVCS, but 54 % of NSCLC and 57 % of SCLC patients went on to relapse at other sites. Of note, 8.8 % of all patients who received RT to the supraclavicular lymph nodes recurred in that area, compared to 33 % of patients who did not receive RT to the supraclavicular area and went on to progress at that site. The median survival of all SVCS patient included in the review was 5.5 months, 1-year overall survival (OS) was 24 %, and 5-year OS was 9 % (Armstrong et al. 1987).

A meta-analysis of prospective and retrospective studies from 1983 to 1997 found that, for patients with SCLC, radiation alone provided a 77.6 % rate of complete SVCS symptom relief and combined chemoradiation resulted in an 83.3 % symptom relief rate. In NSCLC patients, the symptom relief rate was 63.0 % for radiation alone versus 31.3 % with combined chemoradiation (Rowell and Gleeson 2001).

A Belgian experience treating 34 SVCS patients from 1986 to 1993 has been reported. Based on the patient’s performance status, the obstructing mass was treated with doses ranging from 30 Gy in 9 fractions to 54 Gy in 24 fractions. Patients with poor performance status received 5 fractions of 4 Gy at the onset of treatment, while patients with good performance status received 2 fractions of 4 Gy at treatment onset. After this initial treatment schema, patients were re-evaluated and treated with continued rapid high-dose irradiation or switched to a more conventional 2 Gy per fraction dose regimen. 79 % of patients who received 4 Gy daily responded to therapy compared to 67 % of patients who received 2 Gy daily. Four patients received split course therapy; they did not demonstrate a difference in response rate or survival compared to patients who received continuous therapy. Of note, patients with NSCLC responded to treatment more rapidly than patients with SCLC. In general, patients who responded rapidly to therapy had a better OS (Egelmeers et al. 1996).

There have been no randomized trials comparing RT fractionation schemes, and even retrospective evidence is limited (Chan et al. 1997). The more effective fractionation schemes involve giving relatively large doses of 3–4 Gy for the first 2–5 fractions followed by conventional, 2 Gy fractionation to a total dose of 30–50 Gy (Armstrong et al. 1987; Davenport et al. 1978; Egelmeers et al. 1996). The initial radiation field should encompass gross disease and adjacent lymph node beds, and may be altered during treatment as appropriate given changing symptoms and/or tumor size (Wilson et al. 2007).

### Definitive radiotherapy

For select SVCS, treatment with curative intent may be considered. Among others, those with stage II or III NSCLC, limited stage SCLC, and those with low grade lymphoma may be candidates for definitive single or multi-modality treatment. In these cases practitioners should follow typical protocols for treating the lesion in question, giving definitive doses of radiation as indicated by the tumor type and anatomic location. Treatment teams may wish to consider stent placement prior to definitive RT for symptoms palliation during the initial days of RT. As with any bulky tumor, masses causing SVCS may shrink dramatically during definitive RT, and repeat planning mid-treatment may be indicated. Strategies of starting with initial therapy with higher doses 3–4 Gy for first 2–3 days, followed by resumption of conventional fractionation of 1.8–2 Gy/day to deliver definitive total dose can also be considered (Brady and Perez 2007).

### Consideration of hypofractionated radiation therapy

As discussed above, lung cancer is the most common cause of SVCS, and radiation plays an important role in both palliative and definitive treatment. Several studies have demonstrated that hypofractionated regimens can have equivalent outcomes and toxicity profiles to more conventionally fractionated treatment courses in NSCLC (Sundstrøm et al. 2004; Cheung et al. 2014; Din et al. 2013). Additionally, stereotactic body RT, or SBRT, has been shown to produce very high rates of local tumor control in NSCLC and to be well tolerated and efficacious for a variety of lung malignancies (Robinson et al. 2009; Timmerman et al. 2010).

Often, patients who present with SVCS have a contracted life expectancy due to their underlying disease burden. For these patients, 3–4 weeks of daily treatments may be bothersome and are certainly not practical. In the interest of improving patient convenience while maintaining therapeutic outcomes, a shorter fractionation scheme was developed and used at University Hospital in Amsterdam, The Netherlands. A retrospective study of 39 SVCS patients treated with 2 different hypofractionated RT courses from 1986 to 1992 was performed to compare the efficacy and side effects of the different fractionation schemes. 25 patients received a total of 24 Gy in 3 weekly fractions and 7 patients were treated to a total of 16 Gy in 2 weekly fractions. 56 % of patients treated to 24 Gy had a complete response, while only 28 % of patients treated to 16 Gy had a complete response. 96 % of the patients treated to the 24 Gy had a partial response within 4 weeks of treatment, compared to 71 % of patients treated to 16 Gy. Median OS was higher in the group treated to 24 Gy than in the group treated to 16 Gy, 9 versus 3 months, respectively. The most common side effect of treatment was WHO grade 1 dysphagia, which was experienced by 48 and 43 % of the patients treated to 24 and 16 Gy, respectively, and which resolved in most patients by 3 weeks after treatment (Rodrigues et al. 1993).

From 2000 to 2001 a small, prospective trial tested a hypofractioned RT regimen of 12 Gy delivered in 2 fractions 1 week apart to 23 elderly patients with SVCS. Tumor margins of 1–1.5 cm were used and the supraclavicular area was not treated. 39 % of patients reported partial symptom relief at 5 days after the first fraction, and after the second fraction 74 % of patients reported that their symptoms had entirely disappeared. 22 % of patients reported treatment-associated nausea, 26 % reported WHO grade I–II dysphagia, 17 % reported fatigue, and 17 % reported systemic symptoms such as chest pain, rigors and fevers 12–24 h after the first fraction (Lonardi et al. 2002).

Recently, SBRT has become a well-accepted modality for administering radiation at a variety of anatomical sites. Potential benefits of SBRT include a novel biologic rationale for tumor cell kill as well as a decreased number of treatments, resulting in decreased treatment cost (Timmerman et al. 2007; Timmerman et al. 2014). Only one manuscript discussing SBRT for SVCS is available in the current literature (McKenzie et al. 2013). An 82-year-old man presented with SVCS. Positron emission tomography, computed tomography scan, and biopsy demonstrated NSCLC metastatic to the right paratracheal lymph nodes with no other detectable sites of malignancy. SBRT was used to deliver 50 Gy in 5 fractions to the 2.5 cm mediastinal mass. The patient’s symptoms improved after the second fraction, and he experienced no treatment-related side effects. At 3 months follow-up the patient was dyspnea and chest pain free, but he continued to have visible anterior chest wall collateral vessels (McKenzie et al. 2013). This suggests rapid symptom relief and durability of SBRT in this case.

The patient described in the case report was a good candidate for SBRT because he had no evidence of metastatic or primary disease at the time of presentation, and his obstructing tumor was relatively small. SBRT has been shown to have local control rates of up to 97 % in early stage NSCLC (Timmerman et al. 2010). Given the time necessary to generate an SBRT treatment plan, it is not an ideal treatment method for a patient who needs rapid symptom palliation. However, for patients with potentially curable, reasonably sized tumors that are not extremely radiosensitive to conventional therapy but are located in anatomically favorable locations, SBRT should be included in the list of treatment options.

The literature on hypofractionated RT and SBRT for SVCS is not as robust as that for conventionally fractionated RT. However, given the increase in patient convenience and the decrease in treatment cost associated with hypofractionated RT, this may be an area worth investigating in highly select patients, preferably under the auspice of a clinical trial.

### Consideration of proton therapy

Protons have a unique energy distribution pattern in tissue compared to photons. Exploiting this difference may be beneficial in certain cancer sites, as it may allow for higher dose administration to the tumor site with lower dose to adjacent, healthy tissues (Mitin and Zietman 2014). Thus far, however, there is a paucity of high quality clinical evidence for efficacy of protons versus photons at most disease sites (Olsen et al. 2007; De Ruysscher et al. 2012). Proton centers are very expensive to build, and proton therapy is, at this time, much more costly than equivalent photon therapy (Bekelman and Hahn 2014). Not all patients are good candidates for proton therapy. A scoring system has been proposed to give a framework for prioritizing patients that would benefit from proton therapy (Bekelman et al. 2014).

One experience of treating SVCS with proton therapy has been published. A 66-year-old woman presented with a chemoresistant thymic carcinoid tumor that was invading the cardiac tissue. The patient developed SVCS when the tumor reached 15 cm in diameter on chemotherapy. Because of the intimacy of the tumor and the normal cardiac tissue, the decision was made to use proton therapy in an attempt to spare normal, adjacent tissues. A dose of 74 Gray equivalents (GyE) was delivered in 37 fractions. The patient experienced no acute toxicities, and in the months following proton therapy the tumor shrank to 13 cm. At 2 years follow-up, the tumor had continued to decrease in size and the patient exhibited no late toxicities (Sugawara et al. 2014).

In most cases of SVCS, proton therapy offers minimal benefit over photon therapy in terms of decreased toxicity and increased efficacy. Proton therapy should only be considered in highly select circumstances.

### Radiosensitivity

The most common malignant causes of SVCS are NSCLC, SCLC and lymphoma, but breast cancer, seminoma, malignant thymoma, metastatic colon cancer, metastatic renal cell carcinoma, pancreatic cancer, Kaposi’s sarcoma, acute myelomonocytic leukemia, leiomyosarcoma, esophageal carcinoma, childhood tumors, thyroid cancer and mesothelioma have all been implicated (Armstrong et al. 1987; Chen et al. 1990; Yellin et al. 1990; Schraufnagel et al. 1981; Nicholson et al. 1997).These many types of malignancy cover a range of radiosensitivity, with lymphoma traditionally being regarded as more radiosensitive and renal cell carcinoma less so. With the advent of SBRT and other methods of delivering high doses of radiation, however, fewer and fewer cancers are being considered truly radioresistant (De Meerleer et al. 2014). As previously mentioned, RT for SVCS should generally be delayed until a histologic diagnosis of the cause can be determined (Ampil et al. 2012; Kvale et al. 2007). Usually, determining a histologic diagnosis will be more important to guide chemotherapy than RT. However, as our understanding of the mechanisms of radiosensitivity and radioresistance increases, we may be able to make more rational choices as to which patients receive RT and which receive other modalities as first-line treatment (Willers et al. 2013; Lacombe et al. 2013).

## Non-RT treatment modalities

### Steroids

No trial or review has ever demonstrated symptomatic relief or long-term outcome differences attributable to steroid use (Schraufnagel et al. 1981). However, SVCS treatment algorithms often incorporate steroid use as prophylaxis against radiation-induced edema, and steroids may be indicated if airway edema is present at the time of treatment (Ostler et al. 1997). Recent guidelines suggest that if steroids are used in management of SVCS then they should be of high potency and limited duration (Rowell and Gleeson 2001). Steroids should not be relied upon as a primary treatment for SVCS.

### Chemotherapy

The role of chemotherapy in management of SVCS depends largely on the underlying malignant etiology. For chemo-sensitive diseases such as SCLC, non-Hodgkin lymphoma and germ cell tumors, chemotherapy should be considered a mainstay of treatment (Ostler et al. 1997). Indeed, one trial comparing chemotherapy alone versus chemotherapy followed by radiation for SVCS due to SCLC found no difference in survival between the two groups (Spiro et al. 1983). Another trial, however, found that SCLC patients who received combined chemoradiation had a longer time to symptomatic recurrence compared to patients who received chemotherapy alone (Chan et al. 1997). A meta-analysis of prospective and retrospective studies from 1983 to 1997 found that chemotherapy alone (76.9 %), radiation alone (77.6 %) and chemoradiation (83.3 %) provided similar rates of complete SVCS symptom relief in SCLC patients (Rowell and Gleeson 2001). This same meta-analysis found that the typical time to symptom alleviation in patients with chemo-sensitive disease receiving chemotherapy alone is 1–2 weeks.

For less chemo-sensitive malignancies such as NSCLC, the role of chemotherapy up front is less clear. One trial randomizing NSCLC patients to receive either RT alone or RT plus chemotherapy was closed before full accrual due to increased treatment-associated toxicity in the RT plus chemotherapy arm and no difference in outcomes between the two groups (Pereira et al. 1999). A meta-analysis of prospective and retrospective studies from 1983 to 1997 found that outcomes were significantly worse in treating SVCS in NSCLC patients compared to SCLC patients. The rates of complete symptom relief were similar in chemotherapy alone (59.0 %) versus radiation alone (63.0 %) but were actually worse in combined chemoradiation (31.3 %) (Rowell and Gleeson 2001). It should be noticed that the only prospective study examining combined chemoradiation was the previously mentioned trial that closed accrual early, with only sixteen patients enrolled in the combined chemoradiation arm (Pereira et al. 1999).

A small, prospective trial in the early 1980’s compared chemotherapy followed by consolidative radiation to chemotherapy alone in 28 SCLC patients with SVCS. This study found no difference in SVCS recurrence rates between the two trial arms (Spiro et al. 1983).

It should be noted that most studies examining the role of chemotherapy in SVCS were conducted prior to the advent of targeted biologic therapies. In one case report, metastatic sarcoma causing SVCS was treated with imatinib and subsequent symptom resolution was observed (Maki et al. 2002). Tyrosine kinase inhibitors (TKIs) have been shown to have activity in a variety of malignancies (Funakoshi et al. 2014). In the future, TKIs may play an increasingly large role in SVCS palliation and first line treatment of malignancies where TKI use is indicated. No trial has been conducted in the modern era of targeted therapy to assess the efficacy or tolerability of combined targeted therapy and RT for SVCS.

### Stents

**Fig. 2:**
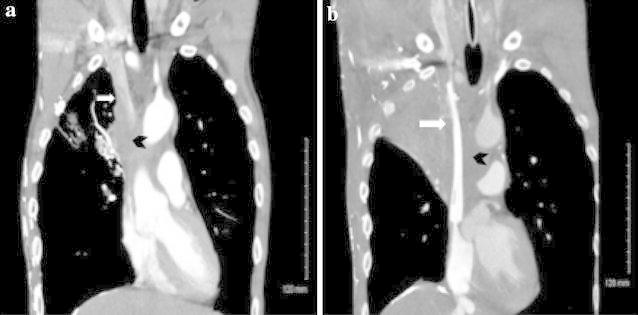
Stent placement for superior vena cava (SVC) syndrome. **a** Pre-stenting: SVC occluded by large tumor. *White arrow* SVC. *Black Chevron* tumor occluding SVC. **b** Post-stenting: SVC now patent. *White arrow* stent in SVC. *Black Chevron* tumor surrounding SVC

Superior vena caval stenting in SVCS was first attempted in 1986. Immediate symptom relief was achieved, but rates of complications and stent migration were initially quite high (Charnsangavej et al. 1986). In the ensuing decades, treatment was refined and new stent materials became available. Today, stent placement is typically done using conscious sedation and local anesthesia, and can be performed as long as the patient can lie flat or semi-supine on the operating table (Watkinson et al. 2008; Charnsangavej et al. 1986). Stent placement is quite effective for symptom relief, with up to 97–99 % of patients experiencing rapid post-operative relief (Maleux et al. 2013; Gwon et al. 2013). Headache is typically relieved immediately after the procedure, facial edema typically resolves within 24 h, and upper extremity and truncal edema may last for up to 72 h after stent placement (Hennequin et al. 1995; Rosch et al. 1992). Figure 2 shows CT images of a patient before and after stent placement, and demonstrates the successful resumption of SVC flow that had previously been completely blocked by a large mediastinal tumor. A recent prospective study found that covered stents retained patency longer than non-covered stents, with a 12-month patency rate of 94 versus 48 % for covered versus non-covered, respectively (Gwon et al. 2013).

In a retrospective study, 164 consecutive SVCS patients receiving stent placement at a single institution from 1992 to 2007 were examined. 12.8 % of patients experienced treatment-associated complications, and 2.4 % died from treatment-associated complications. 21.9 % of patients experienced symptom recurrence, but 75 % of these patients were successfully treated with restenting (Fagedet et al. 2013). This experience is in line with the findings of a 2006 meta-analysis and a 2014 review paper that found percutaneous SVC stenting in the setting of SVCS to have primary patency rates of 64–95 %, secondary patency rates of 93–100 %, recurrence rates 0–40 %, complication rates of 0–25 %, and mortality rates of 3–4 % (Uberoi 2006; Rachapalli and Boucher 2014). Minor complications of stent placement include groin hematoma and local infection at puncture site, while major complications include stent migration, re-occlusions of stents, bleeding, cardiac injury, pulmonary embolism, pulmonary edema, and pericardial tamponade (Rachapalli and Boucher 2014).

Few studies examining hemodynamic changes after stent placement are available. One small study including five patients looked at hemodynamic changes during the first 24 h after surgery. This study found, in all five patients, a decreased pressure gradient across the caval lesion, an increase in pulmonary capillary wedge pressure, and an increase in cardiac output after stent placement. By 24 h post-procedure, the pulmonary capillary wedge pressures and cardiac outputs of the five patients had returned to near-baseline values, while the pressure gradient across the caval lesion continued to decline (Yamagami et al. 2002). All five patients in the hemodynamics study had normal cardiac function, but cases of pulmonary edema and heart failure after stent placement have been reported in the literature (Kishi et al. 1992, 1993). Presumably, these patients had poor cardiac function prior to stent placement, and their cardiac system was not able to accommodate the increased venous return after stent placement (Yamagami et al. 2002). Clinicians should consider carefully before proceeding with stent placement in patients with poor cardiac function.

Although stenting provides significant, rapid relief of SVCS symptoms, it is not usually recommended as first-line treatment in patients with benign disease or in those with long life expectancies, as stent occlusion is likely to occur in the months to years following treatment and stent placement has some intrinsic risk (Uberoi 2006; Rachapalli and Boucher 2014; Watkinson et al. 2008). As therapy improves and the average lifespan of an indwelling stent lengthens, however, some authors have argued that percutaneous stenting may have a role even in benign causes of SVCS (Rizvi et al. 2008). Furthermore, stenting may be used when the maximum dose of radiation has been delivered and SVCS symptoms remain or recur (Rosch et al. 1992).

Randomized studies comparing stents versus RT or chemotherapy are not available. An attempt at doing such a study was made at Princess Margaret Hospital without success (Wilson et al. 2007).

### Radiation therapy and stents

Percutaneously placed stents have become first line treatment for most cases of SVCS. For patients with malignancy-derived SVCS but with a longer life expectancy, RT and/or chemotherapy may be used with curative or palliative intent with or without prior stent placement.

Most patients with SVCS have an underlying malignant cause, and modern treatment usually involves prompt stent placement followed by tissue biopsy and subsequent RT and/or chemotherapy. One study examined 149 cancer patients who received stents for palliation of SVCS between 1993 and 2008. Most of these patients received no treatment before stent placement, but 16.1 % received prior chemotherapy, 2.6 % received prior RT only, and 6.0 % received prior combined chemoradiation. After treatment, 41.6 % received no further treatment, 29 % received chemotherapy alone, 7.4 % received RT alone, and 21.4 % received combined chemoradiation. Patients who received chemotherapy or RT alone before stenting were more likely to die sooner, while patients who received chemoradiation after stenting were more likely to live longer (Lanciego et al. 2009). Although this retrospective study cannot comment on the relative efficacy of different treatment regimens, it seems that, in carefully selected patients, post-stent chemoradiation may have some benefit. At the time of this writing there are no studies comparing long term patency rates of SVC stents in patients who receive RT, chemo, or chemoradiation versus those who do not go on to have further interventions after stent placement. Case reports of SVCS patients receiving further treatment after stent placement suggest that stents may remain patent for months to years following RT or chemoradiation (Hamzik et al. 2015; Komoda et al. 2012).

Stents may be placed after RT. One study found that 80 % of patient who received stents after RT had further symptomatic improvement (Tanigawa et al. 1998).

## Outcomes

The development of SVCS does not necessarily directly impact OS (Sculier et al. 1986). However, in general, SVCS patients have a dismal prognosis. Median survival for all SVCS patients ranges from 1.5 to 10 months (Armstrong et al. 1987; Schraufnagel et al. 1981; Rowell and Gleeson 2001; Marcy et al. 2001). 1-year OS is roughly 24 % and 5-year OS is roughly 9 % (Armstrong et al. 1987). In general, patients with benign or infectious causes of SVCS fare better than do those with malignancies. The average survival of SVCS patients with lung cancer can be as low as 5 months, while for those with mediastinal fibrosis it can be as high as 9 years (Schraufnagel et al. 1981). There have been reports of SVCS patients with NSCLC who have survived up to 9 years after RT and patients with SCLC who have survived up to 13 years after RT (Nogeire et al. 1979; Percarpio and Gray 1979). For patients who have potentially curable disease based on stage and diagnosis, all efforts should be made to provide standard of care definitive therapy. Of note, long-term survivors of SVCS who receive stent placement are at risk for stent migration, an uncommon but potentially deadly complication (Gwon et al. 2013; Martin et al. 2002).

SVCS relapse rates are relatively low after treatment with chemotherapy and/or RT. One meta-analysis found relapse rates of 17 % in SCLC and 19 % in NSCLC (Rowell and Gleeson 2001). Relapse rates following stent placement range from 11 to 12 %. Often, recanalization is possible after stent failure, resulting in long-term stent success rates of greater than 90 % (Rowell and Gleeson 2001; Nagata et al. 2007).

## Areas for future research

Over a decade ago, Rowell and Gleeson published a review on SVCS in which they recommended a randomized trial comparing RT to chemotherapy based on the histology of the underlying malignancy (Rowell and Gleeson 2001). Such a trial has not yet occurred. However, in the past decade significant advances have been made in the treatment of lung cancer. Among other changes, targeted agents and SBRT have come into widespread use in the treatment of lung cancer. Trials are currently exploring the optimal intersection of these modalities in lung cancer in general, and the results of these trials may impact treatment recommendations for SVCS patients.

The published SVCS recurrence rates of almost 20 % after chemotherapy and/or RT are from studies published in 1983–1997 (Rowell and Gleeson 2001). Presumably, treatment outcomes have improved since then, particularly with the advent of combined modality treatments. Due to the relatively rare nature of SVCS, completing large trials is logistically difficult (Wilson et al. 2007). However, trials looking at outcomes with modern therapy would be useful both in counseling patients and in planning therapy. Additionally, trials exploring the benefit of chemotherapy versus targeted therapy and conventional RT versus hypofractionated RT or SBRT should be considered, as the efficacy and side effect profile of these newer treatment modalities is unknown in SVCS.

## Conclusions and recommendations

SVCS causes significant patient distress, usually heralds a serious underlying condition, and necessitates expedient management. Percutaneous stenting can provide rapid symptom relief. Stenting has the additional benefit of not altering local tissue and thus allowing for subsequent biopsy and histologic diagnosis. Both chemotherapy and RT can provide symptom palliation while simultaneously addressing systemic disease. Emergent indications for immediate therapy include laryngeal and cerebral edema and related symptoms, for which endovascular stenting followed by RT should be rapidly initiated.

Most patients who present with SVCS should be offered prompt stent placement for symptom palliation. If the underlying cause of malignancy is not known, this should be elucidated. At the discretion of the treating physicians, chemotherapy and/or RT can then be offered with curative or palliative intent. Some patients may benefit from the increased convenience of a hypofractionated course of RT, although future studies are warranted to further refine treatment guidelines.

## References

[CR1] Ahmann FR (1984). A reassessment of the clinical implications of the superior vena caval syndrome. J Clin Oncol.

[CR2] Ampil F, Caldito G, Previgliano C (2012). Palliative radiotherapy for superior vena caval obstruction by lung cancer: a major issue about timing and a minor issue about efficacy. Ann Thorac Med.

[CR3] Armstrong BA, Perez CA, Simpson JR, Hederman MA (1987). Role of irradiation in the management of superior vena cava syndrome. Int J Radiat Oncol Biol Phys.

[CR4] Baker GL, Barnes HJ (1992). Superior vena cava syndrome: etiology, diagnosis, and treatment. Am J Crit Care.

[CR5] Bekelman JE, Hahn SM (2014). Reference pricing with evidence development: a way forward for proton therapy. J Clin Oncol.

[CR6] Bekelman JE, Asch DA, Tochner Z, Friedberg J, Vaughn DJ, Rash E, Raksowski K, Hahn SM (2014). Principles and reality of proton therapy treatment allocation. Int J Radiat Oncol Biol Phys.

[CR8] Chan RH, Dar AR, Yu E, Stitt LW, Whiston F, Truong P, Vincent MD, Kocha WI (1997). Superior vena cava obstruction in small-cell lung cancer. Int J Radiat Oncol Biol Phys.

[CR9] Charnsangavej C, Carrasco CH, Wallace S, Wright KC, Ogawa K, Richli W, Gianturco C (1986). Stenosis of the vena cava: preliminary assessment of treatment with expandable metallic stents. Radiology.

[CR10] Chen JC, Bongard F, Klein SR (1990). A contemporary perspective on superior vena cava syndrome. Am J Surg.

[CR11] Cheng S (2009). Superior vena cava syndrome: a contemporary review of a historic disease. Cardiol Rev.

[CR12] Cheung P, Faria S, Ahmed S, Chabot P, Greenland J, Kurien E, Mohamed I, Wright JR, Hollenhorst H, de Metz C, Campbell H, Vu TT, Karvat A, Wai ES, Ung YC, Goss G, Shepherd FA, O’Brien P, Ding K, O’Callaghan C (2014). Phase II study of accelerated hypofractionated three-dimensional conformal radiotherapy for stage T1-3 N0 M0 non-small cell lung cancer: NCIC CTG BR.25. J Natl Cancer Inst.

[CR13] Davenport D, Ferree C, Blake D, Raben M (1976). Response of superior vena cava syndrome to radiation therapy. Cancer.

[CR14] Davenport D, Ferree C, Blake D, Raben M (1978). Radiation therapy in the treatment of superior vena caval obstruction. Cancer.

[CR15] de Jager CP, Rutten MJ, Lips DJ (2013). “Benign” superior vena cava syndrome. Intensive Care Med.

[CR16] De Meerleer G, Khoo V, Escudier B, Joniau S, Bossi A, Ost P, Briganti A, Fonteyne V, Van Vulpen M, Lumen N, Spahn M, Mareel M (2014). Radiotherapy for renal-cell carcinoma. Lancet Oncol.

[CR17] De Ruysscher D, Mark Lodge M, Jones B, Brada M, Munro A, Jefferson T, Pijls-Johannesma M (2012). Charged particles in radiotherapy: a 5-year update of a systematic review. Radiother Oncol.

[CR18] Devita V (1997) Cancer: principles and practice of oncology. Lippincott Williams & Wilkins, Philadelphia. ISBN 13: 9780397515738

[CR19] Din OS, Harden SV, Hudson E, Mohammed N, Pemberton LS, Lester JF, Biswas D, Magee L, Tufail A, Carruthers R, Sheikh G, Gilligan D, Hatton MQ (2013). Accelerated hypo-fractionated radiotherapy for non small cell lung cancer: results from 4 UK centres. Radiother Oncol.

[CR20] Egelmeers A, Goor C, van Meerbeeck J, van den Weyngaert D, Scalliet P (1996). Palliative effectiveness of radiation therapy in the treatment of superior vena cava syndrome. Bull Cancer Radiother.

[CR21] Eren S, Karaman A, Okur A (2006). The superior vena cava syndrome caused by malignant disease. Imaging with multi-detector row CT. Eur J Radiol.

[CR22] Fagedet D, Thony F, Timsit JF, Rodiere M, Monnin-Bares V, Ferretti GR, Vesin A, Moro-Sibilot D (2013). Endovascular treatment of malignant superior vena cava syndrome: results and predictive factors of clinical efficacy. Cardiovasc Intervent Radiol.

[CR23] Funakoshi T, Latif A, Galsky MD (2014). Safety and efficacy of addition of VEGFR and EGFR-family oral small-molecule tyrosine kinase inhibitors to cytotoxic chemotherapy in solid cancers: a systematic review and meta-analysis of randomized controlled trials. Cancer Treat Rev.

[CR24] Gauden SJ (1993). Superior vena cava syndrome induced by bronchogenic carcinoma: is this an oncological emergency?. Australas Radiol.

[CR25] Gray BH, Olin JW, Graor RA, Young JR, Bartholomew JR, Ruschhaupt WF (1991). Safety and efficacy of thrombolytic therapy for superior vena cava syndrome. Chest.

[CR26] Gwon DI, Ko GY, Kim JH, Shin JH, Yoon HK, Sung KB (2013). Malignant superior vena cava syndrome: a comparative cohort study of treatment with covered stents versus uncovered stents. Radiology.

[CR27] Hamzik J, Chudej J, Dzian A, Sokol J, Kubisz P (2015). Endovascular stenting in malignant obstruction of superior vena cava. Int J Surg Case Rep.

[CR28] Hennequin LM, Fade O, Fays JG, Bic JF, Jaafar S, Bertal A, Anthoine D, Bernadac PA (1995). Superior vena cava stent placement: results with the Wallstent endoprosthesis. Radiology.

[CR29] Hohloch K, Bertram N, Trumper L, Beissbarth T, Griesinger F (2014). Superior vena cava syndrome caused by a malignant tumor: a retrospective single-center analysis of 124 cases. J Cancer Res Clin Oncol.

[CR30] Hunter W, Johnston W (1757). The history of an aneurysm of the aorta, with some remarks on aneurysms in general.

[CR31] Khan UA, Shanholtz CB, McCurdy MT (2014). Oncologic mechanical emergencies. Emerg Med Clin N Am.

[CR32] Kim HJ, Kim HS, Chung SH (1993). CT diagnosis of superior vena cava syndrome: importance of collateral vessels. AJR Am J Roentgenol.

[CR33] Kishi K, Sonomura T, Mitsuzane K, Nishida N, Yang RJ, Nomura S, Satoh M, Yamada R, Kobayashi H, Juri M (1992). Expandable metallic stent therapy for SVC syndrome-effects on local venous pressure, vascular diameter, symptoms, and these correlations. Nihon Igaku Hoshasen Gakkai Zasshi.

[CR34] Kishi K, Sonomura T, Mitsuzane K, Nishida N, Yang RJ, Sato M, Yamada R, Shirai S, Kobayashi H (1993). Self-expandable metallic stent therapy for superior vena cava syndrome: clinical observations. Radiology.

[CR35] Koetters KT (2012). Superior vena cava syndrome. J Emerg Nurs.

[CR36] Komoda S, Komoda T, Knosalla C, Pavel ME, Morawietz L, von Weikersthal LF, Lehmkuhl HB, Hetzer R (2012). A giant neuroendocrine tumor of the thymus gland causing superior vena cava syndrome. Gen Thorac Cardiovasc Surg.

[CR37] Kvale PA, Selecky PA, Prakash UBS (2007). Palliative care in lung cancer*: Accp evidence-based clinical practice guidelines (2nd edition). Chest.

[CR38] Lacombe J, Mange A, Azria D, Solassol J (2013). Identification of predictive biomarkers to radiotherapy outcome through proteomics approaches. Cancer Radiother..

[CR39] Lacout A, Marcy PY, Thariat J, Lacombe P, El Hajjam M (2012). Radio-anatomy of the superior vena cava syndrome and therapeutic orientations. Diagn Interv Imaging.

[CR40] Lanciego C, Pangua C, Chacon JI, Velasco J, Boy RC, Viana A, Cerezo S, Garcia LG (2009). Endovascular stenting as the first step in the overall management of malignant superior vena cava syndrome. AJR Am J Roentgenol.

[CR41] Lonardi F, Gioga G, Agus G, Coeli M, Campostrini F (2002). Double-flash, large-fraction radiation therapy as palliative treatment of malignant superior vena cava syndrome in the elderly. Support Care Cancer.

[CR42] Maki RG, Awan RA, Dixon RH, Jhanwar S, Antonescu CR (2002). Differential sensitivity to imatinib of 2 patients with metastatic sarcoma arising from dermatofibrosarcoma protuberans. Int J Cancer.

[CR43] Maleux G, Gillardin P, Fieuws S, Heye S, Vaninbroukx J, Nackaerts K (2013). Large-bore nitinol stents for malignant superior vena cava syndrome: factors influencing outcome. AJR Am J Roentgenol.

[CR44] Marcy P-Y, Magne N, Bentolila F, Drouillard J, Bruneton J-N, Descamps B (2001). Superior vena cava obstruction: is stenting necessary?. Support Care Cancer.

[CR45] Martin M, Baumgartner I, Kolb M, Triller J, Dinkel HP (2002). Fatal pericardial tamponade after Wallstent implantation for malignant superior vena cava syndrome. Journal Endovasc Ther.

[CR46] McKenzie JT, McTyre E, Kunaprayoon D, Redmond KP (2013). Stereotactic body radiotherapy for superior vena cava syndrome. Rep Pract Oncol Radiother.

[CR47] Mineo TC, Ambrogi V, Nofroni I, Pistolese C (1999). Mediastinoscopy in superior vena cava obstruction: analysis of 80 consecutive patients. Ann Thorac Surg.

[CR48] Mitin T, Zietman AL (2014). Promise and pitfalls of heavy-particle therapy. J Clin Oncol.

[CR49] Mose S, Stabik C, Eberlein K, Ramm U, Bottcher HD, Budischewski K (2006). Retrospective analysis of the superior vena cava syndrome in irradiated cancer patients. Anticancer Res.

[CR50] Nagata T, Makutani S, Uchida H, Kichikawa K, Maeda M, Yoshioka T, Anai H, Sakaguchi H, Yoshimura H (2007). Follow-up results of 71 patients undergoing metallic stent placement for the treatment of a malignant obstruction of the superior vena cava. Cardiovasc Intervent Radiol.

[CR51] Nicholson AA, Ettles DF, Arnold A, Greenstone M, Dyet JF (1997). Treatment of malignant superior vena cava obstruction: metal stents or radiation therapy. J Vasc Interv Radiol.

[CR52] Nogeire C, Mincer F, Botstein C (1979). Long survival in patients with bronchogenic carcinoma complicated by superior vena caval obstruction. Chest.

[CR53] Olsen DR, Bruland ØS, Frykholm G, Norderhaug IN (2007). Proton therapy: a systematic review of clinical effectiveness. Radiother Oncol.

[CR54] Ostler PJ, Clarke DP, Watkinson AF, Gaze MN (1997). Superior vena cava obstruction: a modern management strategy. Clin oncol (R Coll Radiol).

[CR55] Parish JM, Marschke RF, Dines DE, Lee RE (1981). Etiologic considerations in superior vena cava syndrome. Mayo Clin Proc.

[CR56] Percarpio B, Gray S (1979). Prolonged survival following the superior vena cava syndrome. Chest J.

[CR57] Pereira JR, Martins SJ, Ikari FK, Nikaedo SM, Gampel O (1999). Neoadjuvant chemotherapy vs. radiotherapy alone for superior vena cava syndrome (SVCS) due to non-small cell lung cancer (NSCLC): preliminary results of randomized phase II trial. Eur J Cancer.

[CR7] Perez C, Brady L, Waver D, Freeman C (2007) Perez and Brady’s principles and practice of radiation oncology, 5th edn. Lipincott Williams & Wilkin, Philadelphia. ISBN-13: 9780781763691

[CR58] Rachapalli V, Boucher LM (2014). Superior vena cava syndrome: role of the interventionalist. Can Assoc Radiol J..

[CR59] Rice TW, Rodriguez RM, Light RW (2006). The superior vena cava syndrome: clinical characteristics and evolving etiology. Medicine.

[CR60] Rice TW, Rodriguez RM, Barnette R, Light RW (2006). Prevalence and characteristics of pleural effusions in superior vena cava syndrome. Respirology.

[CR61] Rizvi AZ, Kalra M, Bjarnason H, Bower TC, Schleck C, Gloviczki P (2008). Benign superior vena cava syndrome: stenting is now the first line of treatment. J Vasc Surg.

[CR62] Robinson C, Stephans K, Reddy C, Djemil T, Videtic G (2009). Stereotactic body radiotherapy (SBRT) for radiographically diagnosed primary lung cancer without histologic confirmation. Int J Radiat Oncol Biol Phys.

[CR63] Rodrigues CI, Njo KH, Karim AB (1993). Hypofractionated radiation therapy in the treatment of superior vena cava syndrome. Lung cancer..

[CR64] Rosch J, Uchida BT, Hall LD, Antonovic R, Petersen BD, Ivancev K, Barton RE, Keller FS (1992). Gianturco-Rosch expandable Z-stents in the treatment of superior vena cava syndrome. Cardiovasc Intervent Radiol.

[CR65] Rowell NP, Gleeson FV (2001). Steroids, radiotherapy, chemotherapy and stents for superior vena caval obstruction in carcinoma of the bronchus. Cochrane Database Syst Rev.

[CR66] Schechter MM (1954). The superior vena cava syndrome. Am J Med Sci.

[CR67] Schraufnagel DE, Hill R, Leech JA, Pare JA (1981). Superior vena caval obstruction. Is it a medical emergency?. Am J Med.

[CR68] Sculier JP, Evans WK, Feld R, Deboer G, Payne DG, Shepherd FA, Pringle JF, Yeoh JL, Quirt IC, Curtis JE, Herman JG (1986). Superior vena caval obstruction syndrome in small cell lung cancer. Cancer.

[CR69] Sofue K, Takeuchi Y, Arai Y, Sugimura K (2013). Life-threatening cerebral edema caused by acute occlusion of a superior vena cava stent. Cardiovasc Intervent Radiol.

[CR70] Spiro SG, Shah S, Harper PG, Tobias JS, Geddes DM, Souhami RL (1983). Treatment of obstruction of the superior vena cava by combination chemotherapy with and without irradiation in small-cell carcinoma of the bronchus. Thorax.

[CR71] Stanford W, Jolles H, Ell S, Chiu LC (1987). Superior vena cava obstruction: a venographic classification. AJR Am J Roentgenol.

[CR72] Sugawara K, Mizumoto M, Numajiri H, Ohno T, Ohnishi K, Ishikawa H, Okumura T, Sakurai H (2014) Proton beam therapy for a patient with a giant thymic carcinoid tumor and severe superior vena cava syndrome. Rare Tumors 6(2). doi:10.4081/rt.2014.5177 10.4081/rt.2014.5177 PMC4083663 25002943

[CR73] Sundstrøm S, Bremnes R, Aasebø U, Aamdal S, Hatlevoll R, Brunsvig P, Johannessen DC, Klepp O, Fayers PM, Kaasa S (2004). Hypofractionated palliative radiotherapy (17 Gy per two fractions) in advanced non-small-cell lung carcinoma is comparable to standard fractionation for symptom control and survival: a National Phase III Trial. J Clin Oncol.

[CR74] Taguchi J, Kinoshita I, Akita H (2011). Gan to kagaku ryoho [Superior vena cava syndrome]. Cancer Chemother.

[CR75] Tanigawa N, Sawada S, Mishima K, Okuda Y, Mizukawa K, Ohmura N, Toita T, Ogawa K, Kobayashi M, Kobayashi M (1998). Clinical outcome of stenting in superior vena cava syndrome associated with malignant tumors. Comparison with conventional treatment. Acta Radiol..

[CR76] Timmerman RD, Kavanagh BD, Cho LC, Papiez L, Xing L (2007). Stereotactic body radiation therapy in multiple organ sites. J Clin Oncol.

[CR77] Timmerman R, Paulus R, Galvin J, Michalski J, Straube W, Bradley J, Fakiris A, Bezjak A, Videtic G, Johnstone D, Fowler J, Gore E, Choy H (2010). Stereotactic body radiation therapy for inoperable early stage lung cancer. JAMA J Am Med Assoc.

[CR78] Timmerman RD, Herman J, Cho LC (2014). Emergence of stereotactic body radiation therapy and its impact on current and future clinical practice. J Clin Oncol.

[CR79] Trigaux JP, Van Beers B (1990). Thoracic collateral venous channels: normal and pathologic CT findings. J Comput Assist Tomogr.

[CR80] Uberoi R (2006). Quality assurance guidelines for superior vena cava stenting in malignant disease. Cardiovasc Intervent Radiol.

[CR81] Wan JF, Bezjak A (2010). Superior vena cava syndrome. Hematol Oncol Clin N Am.

[CR82] Watkinson AF, Yeow TN, Fraser C (2008). Endovascular stenting to treat obstruction of the superior vena cava. BMJ (Clinical research ed).

[CR83] Willers H, Azzoli CG, Santivasi WL, Xia F (2013). Basic mechanisms of therapeutic resistance to radiation and chemotherapy in lung cancer. Cancer J..

[CR84] Wilson LD, Detterbeck FC, Yahalom J (2007). Superior Vena Cava Syndrome with malignant causes. N Engl J Med.

[CR85] Wilson P, Bezjak A, Asch M, Barton R, Wong R, Levin W, Kane G, Kirkbride P (2007). The difficulties of a randomized study in superior vena caval obstruction. J Thorac Oncol.

[CR86] Yamagami T, Nakamura T, Kato T, Iida S, Nishimura T (2002). Hemodynamic changes after self-expandable metallic stent therapy for vena cava syndrome. AJR Am J Roentgenol.

[CR87] Yellin A, Rosen A, Reichert N, Lieberman Y (1990). Superior vena cava syndrome. The myth–the facts. Am Rev Respir Dis.

[CR88] Yu JB, Wilson LD, Detterbeck FC (2008). Superior Vena Cava Syndrome: a proposed classification system and algorithm for management. J Thorac Oncol.

